# Standalone 29-MHz micro-ultrasound for classifying clinically significant prostate cancer: a systematic review and diagnostic test accuracy meta-analysis of prospective studies

**DOI:** 10.1007/s00261-025-05218-x

**Published:** 2025-10-31

**Authors:** Ahmed M. Abdel Gawad, Ahmed Y. Aboelsaad, Ahmed Fawzi Elsayed, Elsayed Mohamed Abd El-Hamid Hassan, Ahmed Yahia Ashour, Alshimaa Yahia Ashour, Eman M. El-Dydamony, Maha M. Elzamek, Amany Ahmed Soliman, Hany Elsegeay, Ahmed Farag wahsh, Mohamed Fathy Elebiary, Mohamed Abd El Rahman Alkenawy, Mohamed Hamouda Elkasaby, Atef A. Hassan

**Affiliations:** 1 Urology Department, Faculty of Medicine, Al-Azhar University, Damietta, Egypt; 2 Radiology Department, Faculty of Medicine, Al-Azhar University, Damietta, Egypt; 3 Radiology Department, Faculty of Medicine for Girls, Al-Azhar University, Cairo, Egypt; 4 Urology department, Faculty of medicine for girls, Al-Azhar University, Cairo, Egypt; 5 Urology Department, Faculty of Medicine, Al- Azhar university, Asyut, Egypt; 6 Urology Department, Faculty of Medicine, Al-Azhar University, Cairo, Egypt; 7 Radiology Department, Faculty of Medicine, Al-Azhar University,, Cairo, Egypt; 8 Faulty of Medicine, Al-Azhar University, Cairo, Egypt

**Keywords:** Micro-ultrasound, Clinically significant prostate cancer, PRI-MUS, Diagnostic test accuracy, HSROC, Likelihood ratios, Fagan nomogram

## Abstract

**Background:**

Micro-ultrasound (micro-US; 29-MHz) offers real-time, high-resolution prostate imaging, but its stand-alone diagnostic accuracy remains uncertain. We synthesized prospective evidence to evaluate micro-US for classifying clinically significant prostate cancer (csPCa) using histopathology as the reference standard.

**Methods:**

We searched PubMed, Embase, Scopus, and Web of Science (inception–20 May 2025) for prospective studies assessing micro-US as an index test on a diagnostic pathway. Data were pooled using random-effects models on logit-transformed sensitivity and specificity, with an HSROC representation and model diagnostics. Subgroup and meta-regression analyses explored heterogeneity, including threshold (PRI-MUS) and spectrum effects. Clinical utility was appraised using Fagan nomograms and a likelihood-ratio scatter. Small-study effects were evaluated with Deeks’ test.

**Results:**

Five prospective studies met criteria. Pooled sensitivity was 0.84 (95% CI 0.65–0.94) and pooled specificity was 0.41 (95% CI 0.25–0.59), indicating moderate discrimination on HSROC. Secondary metrics were concordant (PLR 1.45, 95% CI 1.17–1.80; NLR 0.37, 95% CI 0.23–0.61; DOR 3.95, 95% CI 2.48–6.30). On a 25% pre-test probability, the Fagan nomogram showed modest shifts (~ 33% after a positive test; ~11% after a negative), supporting a triage/rule-out role. Heterogeneity was substantial and strongly influenced by threshold and clinical spectrum differences; subgroup and meta-regression suggested that spectrum-related factors were associated with lower specificity, whereas no covariate robustly altered sensitivity (exploratory given small k). Model checks were acceptable, and Deeks’ test showed no evidence of small-study effects (*p* ≈ 0.70).

**Conclusion:**

As a stand-alone index test for csPCa classification, micro-US demonstrates high sensitivity but low specificity, yielding modest impact on post-test probability. These findings support micro-US as a complementary/triage (rule-out) adjunct, particularly when mpMRI is unavailable, contraindicated, or delayed, while highlighting the need for standardized PRI-MUS thresholds, reader training, and larger multicenter studies to refine specificity and clarify integration with MRI-based pathways.

**Supplementary Information:**

The online version contains supplementary material available at 10.1007/s00261-025-05218-x.

## Introduction

Prostate cancer (PCa) is one of the most frequently diagnosed malignancies among men and remains a leading cause of cancer-related mortality worldwide [1]. In 2020, over 1.4 million new cases were diagnosed globally, with PCa accounting for approximately 375,000 deaths [2]. Despite advances in screening and management, early detection and accurate risk stratification of prostate cancer remain major clinical challenges [3].

The standard diagnostic approach for PCa often begins with prostate-specific antigen (PSA) testing; however, the limited specificity of PSA contributes to a high rate of unnecessary biopsies, increased patient anxiety, and potential adverse effects [4]. Transrectal ultrasound (TRUS)-guided systemic biopsy has traditionally been used to obtain prostate tissue samples for diagnosis [5]. Nevertheless, this technique is invasive and presents notable drawbacks, including suboptimal sensitivity and a considerable risk of sampling error [6]. Due to these limitations, TRUS alone is not considered a reliable tool for detecting PCa, and recent clinical guidelines recommend the exploration of alternative imaging modalities that can improve lesion visualization and enable more accurate, targeted prostate biopsies [7].

Among these, micro-ultrasound (micro-US) has emerged as a novel technique that operates at a high frequency of 29 MHz, providing image resolution of up to 70 micrometers, thereby enabling enhanced visualization of prostate tissue abnormalities [8]. Micro-US represents a novel, high-resolution imaging technology offering real-time visualization of prostate tissue with significantly improved spatial resolution compared to conventional TRUS [9]. Despite growing clinical interest and several prospective studies investigating micro-US, its standalone diagnostic performance remains variably reported, with no prior meta-analysis focusing exclusively on its accuracy as an independent imaging modality.

While previous meta-analyses have broadly assessed various ultrasound modalities, a focused quantitative synthesis evaluating the standalone diagnostic accuracy of 29 MHz micro-ultrasound is currently lacking. This approach is critical, as pooling data from technologically heterogeneous platforms can obscure the true performance of a specific modality. Prior reviews have summarized micro-US alongside other ultrasound techniques and/or in comparison with mpMRI. In particular, DuBois et al., 2025 offered a comprehensive narrative comparison of micro-US and mpMRI but did not provide pooled bivariate diagnostic-accuracy estimates and included heterogeneous ultrasound modalities [10]. Our contribution is a **prospective-only**,** ExactVu-only**,** stand-alone DTA synthesis** with HSROC and moderator analyses. This study provides modality-specific performance estimates based exclusively on 29-MHz micro-ultrasound. This study aims to fill that evidence gap by providing precise, modality-specific performance estimates based exclusively on prospective studies of 29 MHz micro-ultrasound.

## Methods

This systematic review and meta-analysis adhered to the guidelines outlined in the Cochrane Handbook for systematic reviews and meta-analyses of diagnostic test accuracy (DTA) studies[11] and followed the PRISMA statement guidelines [12].

### Searching, Screening, and study selection

A comprehensive search was conducted in PubMed (MEDLINE), Embase, Scopus, and Web of Science Core Collection from inception to 20 May 2025, with no language or study-design limits at the search stage. The strategy combined controlled vocabulary and free-text terms for prostate cancer and micro-ultrasound, for example: (prostate cancer OR *Prostatic Neoplasms*[MeSH] OR prostatic neoplasm*) AND (micro-ultrasound OR microultrasound OR “micro ultrasound” OR 29 MHz OR 29-MHz OR 29 MHz OR ExactVu OR “Exact Imaging” OR PRI-MUS OR PRIMUS); database-specific subject headings and syntax were applied, and full strategies are provided in Supplementary Table 1.

Search results were de-duplicated in EndNote version 12, and two reviewers independently screened titles/abstracts and full texts against prespecified eligibility [A.Y.A and A.F.E], with disagreements resolved by a third reviewer [A.A.H]. We also performed backward/forward citation chasing of included studies and key reviews and contacted authors when necessary to obtain per-patient 2 × 2 data. Study selection is summarized in a PRISMA flow diagram.

### Eligibility criteria

We included prospective, peer-reviewed studies of adult men undergoing evaluation for suspected prostate cancer on a diagnostic pathway (biopsy-naïve or prior-negative) in which 29-MHz micro-ultrasound was performed as a standalone diagnostic test with lesion- or patient-level classification (e.g., PRI-MUS), and in which histopathology from systematic and/or targeted biopsy obtained in the same diagnostic episode served as the reference standard (whole-mount radical prostatectomy was accepted only when participants were enrolled before the biopsy decision). Studies had to report, or allow derivation of, per-patient true positives, false positives, true negatives, and false negatives at a pre-specified positivity threshold (primary: PRI-MUS ≥ 3; secondary: ≥4) or provide sensitivity and specificity with a sample size enabling bivariate pooling. The acceptable reference standard was contemporaneous histopathology from the same diagnostic episode (systematic and/or targeted prostate biopsy). Whole-mount radical prostatectomy was accepted **only** when participants were enrolled before the biopsy decision. By contrast, we excluded studies using non-histopathology references (e.g., mpMRI/PI-RADS, PSMA PET/CT, or other imaging), prior or remote biopsy results outside the current work-up, clinical follow-up/PSA kinetics without biopsy, composite consensus references, or partial-verification designs (biopsy performed only in micro-US–positive lesions).

We excluded cohorts composed of biopsy-proven cancer imaged after diagnostic biopsy; men with prior definitive prostate treatment (radical prostatectomy, radiotherapy, focal therapy, androgen-deprivation therapy) or prior TURP/HoLEP; retrospective designs; studies using a non-histopathology reference (e.g., prior biopsy as “truth,” imaging alone, or clinical follow-up); reviews, editorials, conference abstracts, animal studies; and reports lacking sufficient data for per-patient diagnostic-accuracy estimates. Variability in biopsy sampling (systematic/targeted/combined), guidance modality (TRUS/micro-US/MRI-fusion), biopsy approach (transrectal/transperineal), MRI status, and csPCa definition (e.g., ISUP ≥ 2) was not used as an exclusion criterion; these factors were coded a priori and examined via meta-regression and subgroup analyses.

### Data extraction

Two reviewers independently piloted and then used a standardized extraction form [A.F.E and M.F.E]; discrepancies were resolved by consensus or a third reviewer [A.A.H]. For each eligible study, we extracted: setting (country/region), study design, sample size, patient population (e.g., suspected PCa, prior-negative biopsy), biopsy history (biopsy-naïve vs. prior-negative, proportion), median PSA (ng/mL), median prostate volume (cc), index test/device (29-MHz micro-ultrasound; ExactVu), biopsy method (e.g., MRI/micro-US targeted, systematic), prostate cancer prevalence, csPCa definition (e.g., GG ≥ 2 or GS ≥ 3 + 4 with core length criteria), and diagnostic performance (sensitivity and specificity). Where available, we recorded the positivity threshold used for micro-US classification (e.g., PRI-MUS ≥ 3 or ≥ 4) that underpinned reported accuracy. When sensitivity/specificity were reported without full 2 × 2 data, we reconstructed per-patient TP/FP/TN/FN using denominators provided; if totals were insufficient, we contacted authors.

### Risk of bias assessment

The risk of bias in included studies was evaluated using the Quality Assessment for Diagnostic Accuracy Studies 2 (QUADAS-2) checklist [13]. This assessment considered four main domains: patient selection, index test, reference standard, and flow and timing, alongside applicability concerns for all domains.

### Statistical analysis

All analyses were performed in **Python** (pandas, NumPy, SciPy, statsmodels, matplotlib) using custom code. We meta-analyzed **logit-transformed sensitivity and specificity** with a **DerSimonian–Laird random-effects** model to obtain pooled estimates and 95% CIs, and quantified heterogeneity with **Cochran’s Q** and **I²**. A **hierarchical summary ROC (HSROC)** representation was obtained by **weighted least squares** regression of logit(sensitivity) on logit(false-positive rate), from which we plotted the smooth curve and the model-based summary operating point. **DOR**,** PLR**,** and NLR** were pooled via random-effects on **log(DOR)**, **log(PLR)**, and **log(NLR)**, respectively. Small-study effects were assessed with **Deeks’ funnel plot** implemented as a linear regression of **1/√(effective sample size)** on **log(DOR)** [14]. To explore heterogeneity, we ran **prespecified subgroup analyses** (e.g., sample size, setting, biopsy approach, prior-negative biopsy proportion, prevalence) using group-wise random-effects on logit metrics, and **meta-regression** (weighted least squares) with continuous and categorical covariates. **Threshold effects** were evaluated by plotting **logit(sensitivity)** versus **logit(specificity)** and estimating their correlation; distributional and residual checks were inspected to identify influential patterns. For clinical utility, we used a **Fagan nomogram** with a **25% pre-test probability**, chosen a priori to approximate typical biopsy-referral prevalence in contemporary pathways.

## Results

### Study selection and characteristics

**Fig. 1:**
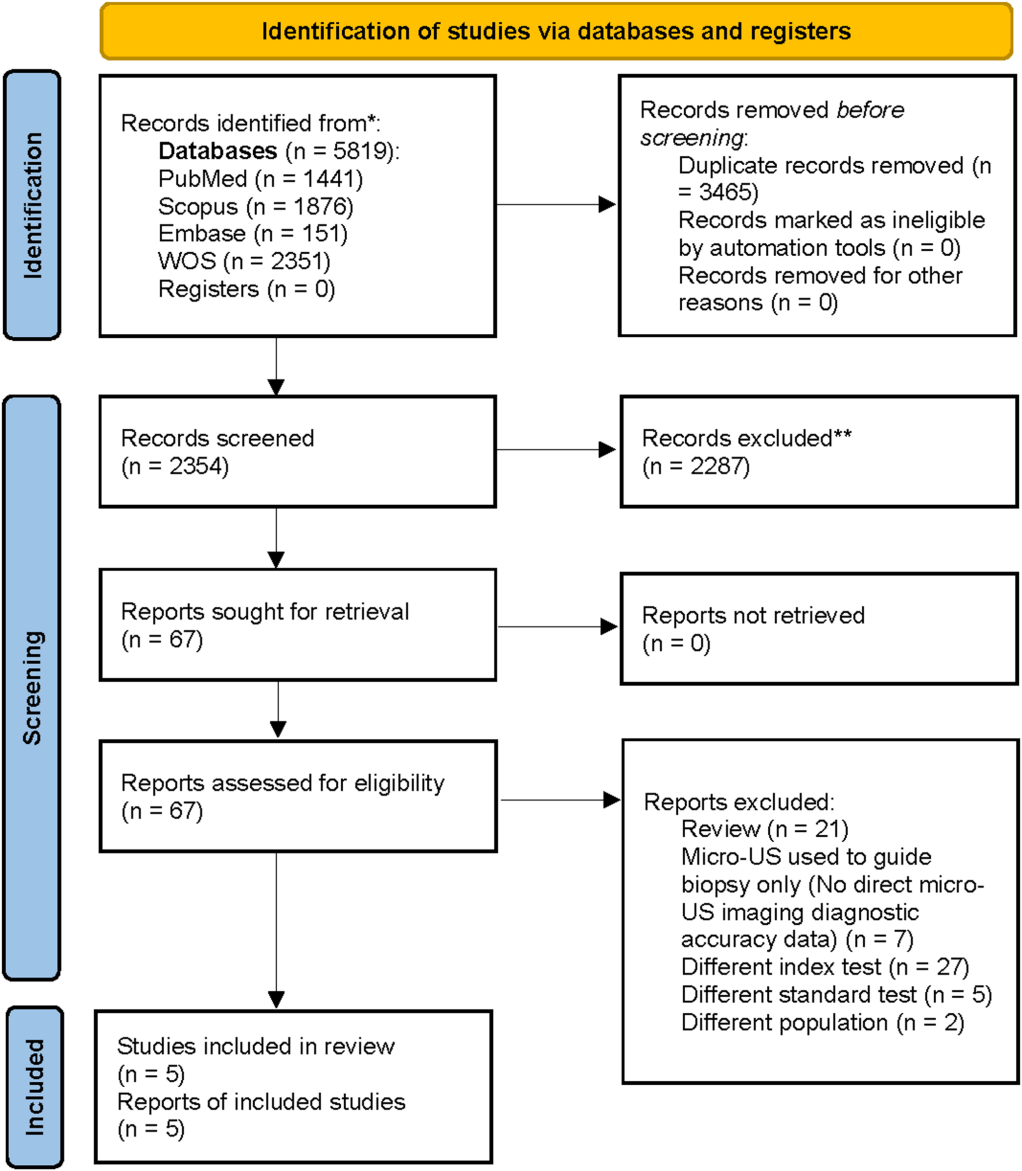
PRSMA flow diagram

The search identified **5**,**819** records; after removing **3**,**465** duplicates, **2**,**354** titles/abstracts were screened. **67** full texts were reviewed and **62** were excluded (review articles; micro-US used only to guide biopsy without standalone diagnostic accuracy; different index test; different reference standard; different population). **Five** prospective studies met the criteria and were included in the quantitative synthesis: **Avolio 2025**,** Klotz 2020**,** Lughezzani 2021**,** Lopci 2021**, and **Pavlovich 2020** (Fig. 1).

All studies assessed **29-MHz micro-ultrasound (ExactVu)**. Four were diagnostic-pathway cohorts of men with **suspected** prostate cancer; **Lopci 2021** represented a **spectrum-selected** cohort (persistently elevated PSA with negative/contra mpMRI), and **Pavlovich 2020** was a **randomized guidance** trial from which per-patient accuracy could be derived. PRI-MUS thresholds varied (≥ 3 or ≥ 4). Across studies, sensitivity for clinically significant cancer ranged from **0.60 to 0.94**, while specificity ranged from **0.22 to 0.63** (Table 1).

**Table 1:** Characteristics and diagnostic performance of micro-ultrasound in prospective studies

Variable	Avolio et al. [15]	Klotz et al. [9]	Lopci et al. [20]	Lughezzani et al. [19]	Pavlovich et al. [16]
Setting	Italy	North America and Europe	Italy	Italy	USA
Study Design	Prospective	Prospective	Prospective	Prospective	Prospective
Sample Size	1423	1040	20	320	837
Patient Population	Men with suspected PCa	Men with suspected PCa	Men with prior negative biopsy	Men with suspected PCa and PI-RADS ≥ 3 lesion	Men with indication for biopsy
Biopsy History	28% prior negative biopsy	Mixed (34% prior biopsy)	100% prior negative biopsy	37.5% prior negative biopsy	Biopsy-naïve
Median PSA (ng/mL)	7.0	7.0	7.0	7.3	6.0
Median Prostate Volume (cc)	50	38	Not Reported	45	< 60 cc
Index Test/Device	Micro-US/Exact Vu	Micro-US/Exact Vu	Micro-US/Exact Vu	Micro-US/Exact Vu	Micro-US/Exact Vu
Biopsy Method	MRI/micro-US targeted…	MRI/micro-US targeted…	PSMA-PET/TRUS fusion…	MRI/micro-US targeted	micro-US targeted/systematic
Prostate Cancer Prevalence	0.36	0.61	0.30	0.363	0.663
csPCa Definition	GS ≥ 3 + 4 & ≥4 mm	Gleason Grade Group ≥ 2	Gleason Score ≥ 7	Gleason Score ≥ 7	GG ≥ 2 or > 50% core involvement
Sensitivity	0.85	0.94	0.80	0.897	0.60
Specificity	0.53	0.22	0.53	0.26	0.632

**cc**, cubic centimeters; **csPCa**, clinically significant prostate cancer; **GG**, Gleason Grade Group; **GS**, Gleason Score; **Micro-US**, micro-ultrasound; **MRI**, magnetic resonance imaging; **ng/mL**, nanograms per milliliter; **PCa**, prostate cancer; **PI-RADS**, Prostate Imaging Reporting and Data System; **PSA**, prostate-specific antigen; **PSMA-PET**, prostate-specific membrane antigen positron emission tomography; **TRUS**, transrectal ultrasound

### Risk of bias using QUADAS-2 tool

QUADAS-2 appraisal showed **low risk** for the index test, reference standard, and flow/timing domains in most studies, with **some concerns** most commonly in **patient selection** because of spectrum enrichment (e.g., MRI-negative or referral filters) and, for Pavlovich 2020, the randomized guidance design (Supplementary Fig. 1).

### Pooled diagnostic performance and heterogeneity

**Fig. 2:**
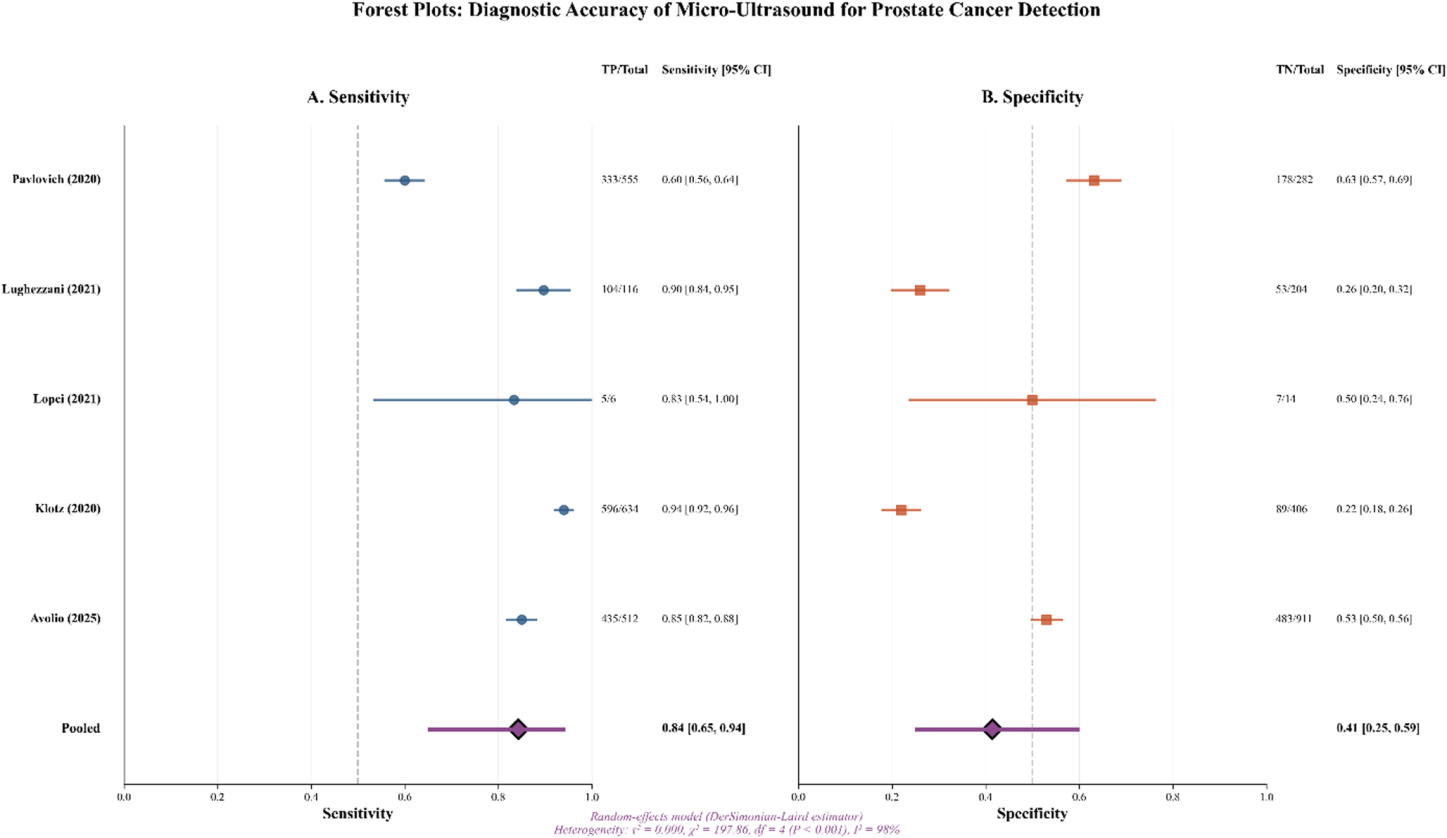
Forest plot of Sensitivity and Specificity

**Fig. 3:**
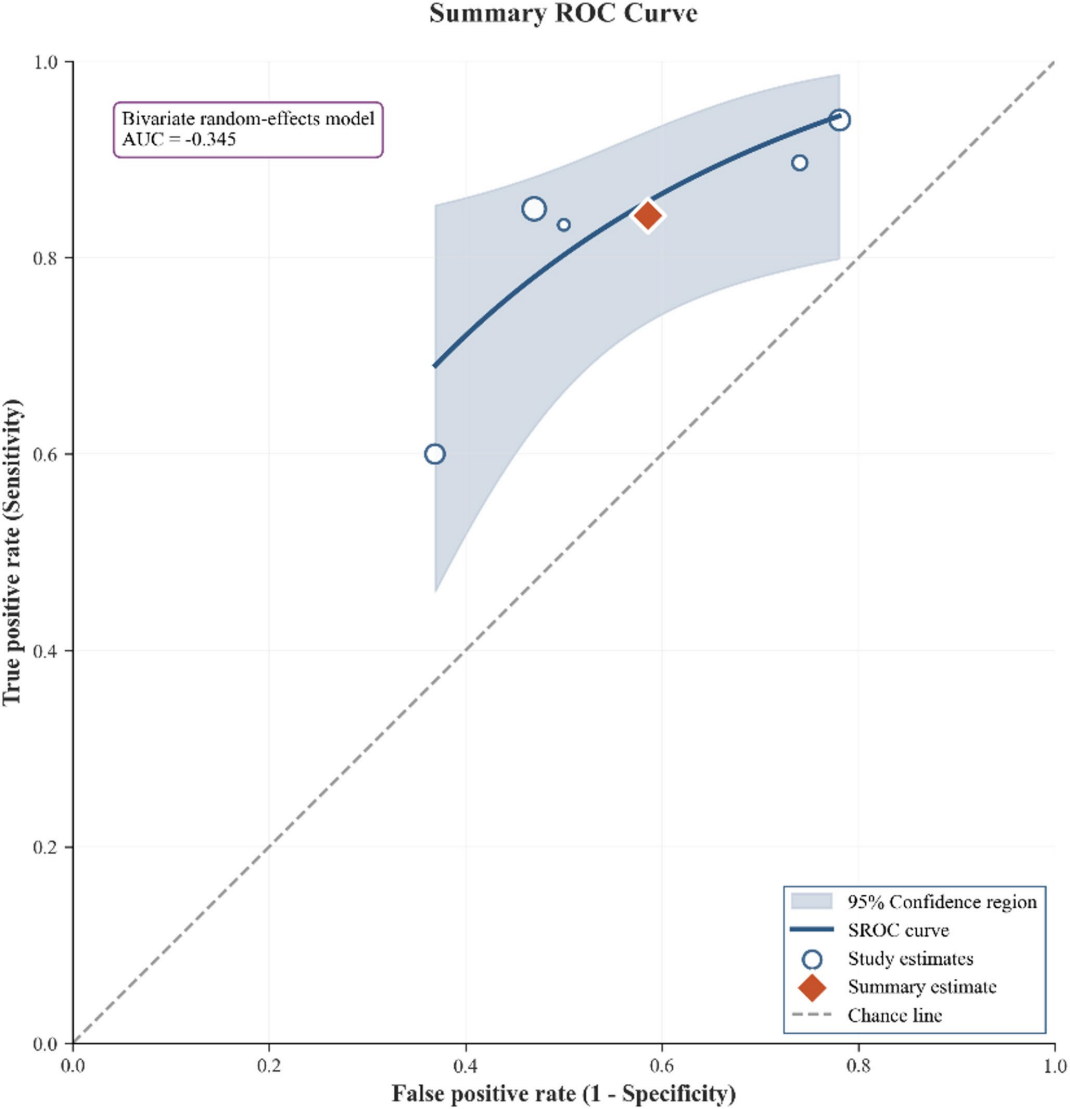
HSROC Curve

**Fig. 4:**
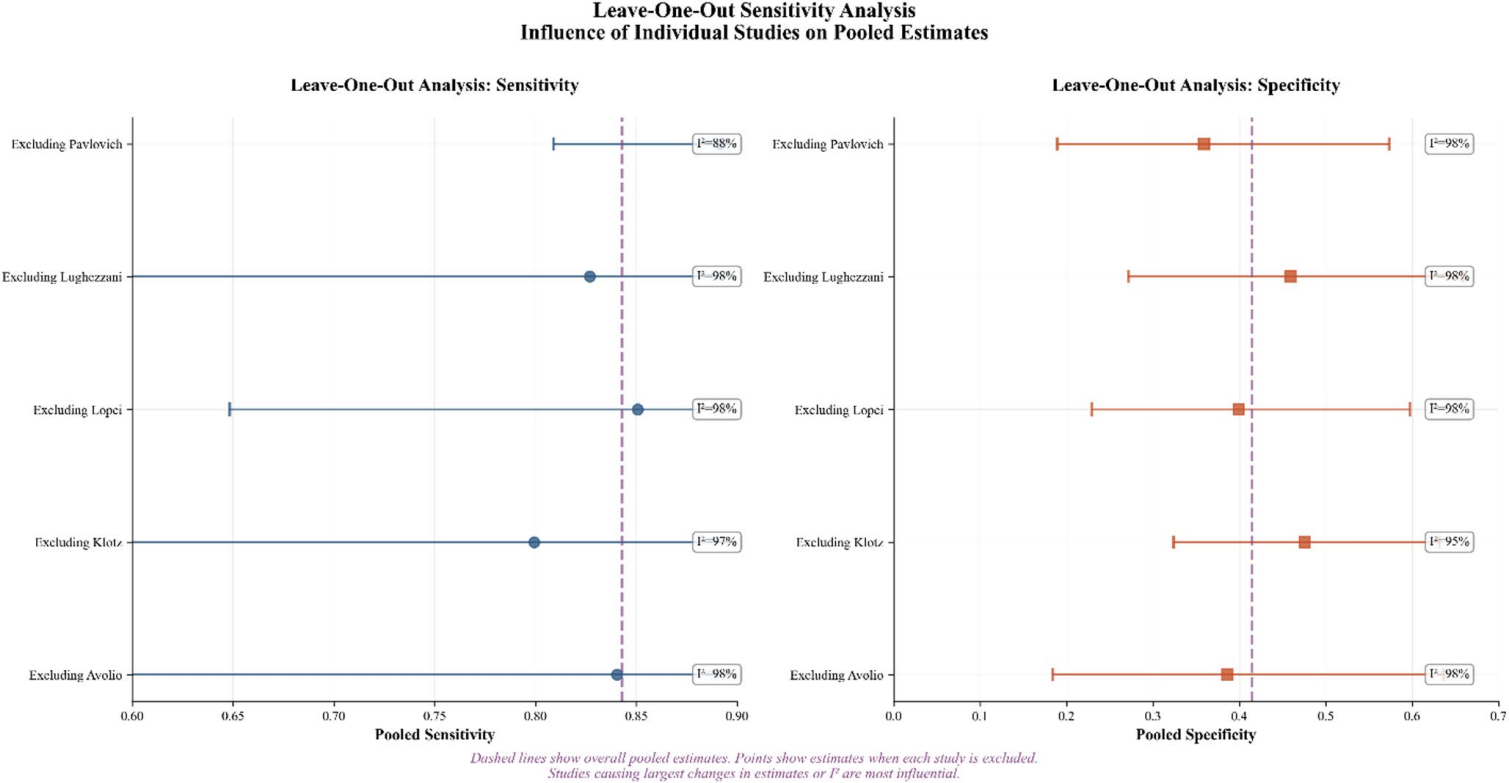
Leave-One-Out Analysis

The random-effects model on logit-transformed sensitivity and specificity (Fig. 2) yielded a **pooled sensitivity of 0.84 (95% CI 0.65–0.94)** and a **pooled specificity of 0.41 (95% CI 0.25–0.59)** for classifying clinically significant prostate cancer. Between-study heterogeneity was **high**, consistent with variations in clinical spectrum, thresholds (PRI-MUS ≥ 3 vs. ≥ 4), and inclusion of a guidance trial. The HSROC (Fig. 3) indicates **moderate** discrimination, with the summary operating point near the study cloud, reflecting threshold trade-offs. Leave-one-out analyses (Fig. 4) showed **no single study**, including Pavlovich 2020 or Lopci 2021, materially altered pooled sensitivity or specificity; estimates shifted within their confidence bands. The bivariate boxplot (Supplementary Fig. 2) showed most studies within the central region with some peripheral points. A strong **negative correlation** between **logit(sensitivity)** and **logit(specificity)** (Pearson *r* ≈ − **0.916**, *R²* ≈ **0.838**, *p* ≈ **0.029**; Supplementary Fig. 3) supports a **threshold effect** as a principal driver of variability. The likelihood-ratio scatter (Supplementary Fig. 4) places the summary estimate in a **rule-out-leaning** zone (PLR < 10; NLR around 0.3–0.4). **Sensitivity analyses** excluding the **guidance trial** (Pavlovich 2020) and the **spectrum-selected** cohort (Lopci 2021) produced pooled estimates that remained **within the primary model’s 95% CIs.**

### Subgroup and meta-regression findings

Across subgroups, sensitivity remained broadly similar while specificity varied with clinical spectrum and design. Single-center and multi-center studies showed comparable pooled sensitivity (0.78 vs. 0.75) and specificity (0.42 vs. 0.48), both with substantial residual heterogeneity. Larger samples (≥ 500) yielded sensitivity 0.77 and specificity 0.46, whereas smaller studies had wide intervals and imprecise specificity, reflecting sparse data. Populations enriched by prior negative biopsy or high pre-test risk performed similarly to mixed “suspected PCa” cohorts for sensitivity but tended to show lower and more variable specificity. Categories represented by a single study (biopsy-naïve only; targeted-only biopsy; “screening” population; high prevalence ≥ 50%) consistently displayed very high sensitivity paired with **very low specificity** (e.g., specificity reported as ~ 0.00–0.15), underscoring a threshold/spectrum effect and the need for cautious interpretation of these isolated strata. Overall, subgroup contrasts did not reveal a stable “high-specificity” configuration for micro-US; where specificity improved modestly (e.g., lower-prevalence settings), confidence intervals overlapped extensively (Table 2).

**Table 2:** Subgroup analysis of diagnostic accuracy

Parameter	Category	*N* Studies	Sensitivity [95% CI]	Specificity [95% CI]	I² (%)
Study Setting	Single-center	3	0.78 [0.69, 0.86]	0.42 [0.25, 0.61]	78
	Multi-center	2	0.75 [0.65, 0.83]	0.48 [0.28, 0.69]	85
Sample Size	Large (≥ 500)	3	0.77 [0.69, 0.84]	0.46 [0.32, 0.61]	85
	Small (< 500)	2	0.76 [0.55, 0.90]	0.28 [0.01, 0.82]	0
Biopsy History	Mixed/Prior negative	4	0.77 [0.70, 0.84]	0.44 [0.31, 0.58]	82
	Biopsy-naïve	1	0.76 [Single study]	0.15 [Single study]	0
Population Type	Suspected PCa/High-risk	4	0.78 [0.70, 0.85]	0.44 [0.30, 0.59]	80
	Screening	1	0.76 [Single study]	0.15 [Single study]	0
Biopsy Method	Systematic only	2	0.75 [0.65, 0.83]	0.48 [0.28, 0.69]	85
	Combined approach	2	0.77 [0.66, 0.86]	0.42 [0.20, 0.67]	85
	Targeted only	1	0.90 [Single study]	0.00 [Single study]	0
PCa Prevalence	Low (< 50%)	4	0.76 [0.69, 0.82]	0.46 [0.33, 0.60]	72
	High (≥ 50%)	1	0.90 [Single study]	0.00 [Single study]	0

Meta-regression supported these patterns. No covariate significantly shifted sensitivity. By contrast, several covariates were associated with lower specificity. A higher proportion of men with a prior negative biopsy independently predicted lower specificity (coefficient − 0.042, 95% CI − 0.079 to − 0.005, *p* = 0.027; R² = 0.72), consistent with spectrum enrichment. Being a screening-type cohort was also associated with lower specificity (coefficient − 1.267, 95% CI − 2.200 to − 0.334, *p* = 0.008; R² = 0.78). Increasing prevalence showed a borderline association with lower specificity (coefficient − 0.045, 95% CI − 0.092 to 0.002, *p* = 0.061), again aligning with spectrum effects. Median PSA, study size, center type, and combined vs. systematic biopsy method were not significant moderators of either sensitivity or specificity. These findings reinforce that heterogeneity is driven primarily by **spectrum/threshold phenomena** rather than by procedural particulars alone. Given the small number of studies (k = 5), this meta-regression results should be interpreted as exploratory and hypothesis-generating (Table 3).

**Table 3:** Meta-regression analysis exploring sources of heterogeneity

Covariate	Outcome	Coefficient	SE	95% CI	*P*-value	*R*²
Median PSA (ng/mL)	Sensitivity	0.045	0.089	[−0.130, 0.220]	0.612	0.08
	Specificity	−0.156	0.142	[−0.434, 0.122]	0.272	0.31
PCa Prevalence (%)	Sensitivity	0.012	0.015	[−0.017, 0.041]	0.423	0.19
	Specificity	−0.045	0.024	[−0.092, 0.002]	0.061	0.68
Prior Negative Biopsy (%)	Sensitivity	0.008	0.012	[−0.015, 0.031]	0.501	0.13
	Specificity	−0.042	0.019	[−0.079, −0.005]	0.027	0.72
Log Sample Size	Sensitivity	−0.089	0.156	[−0.395, 0.217]	0.568	0.10
	Specificity	0.234	0.249	[−0.254, 0.722]	0.348	0.25
Multi-center Study	Sensitivity	−0.156	0.298	[−0.740, 0.428]	0.601	0.08
	Specificity	0.267	0.476	[−0.666, 1.200]	0.575	0.09
Combined Biopsy Method	Sensitivity	0.089	0.298	[−0.495, 0.673]	0.766	0.03
	Specificity	−0.267	0.476	[−1.200, 0.666]	0.575	0.09
Screening Population	Sensitivity	−0.156	0.298	[−0.740, 0.428]	0.601	0.08
	Specificity	−1.267	0.476	[−2.200, −0.334]	0.008	0.78

### Secondary metrics, clinical impact, and model adequacy

**Fig. 5:**
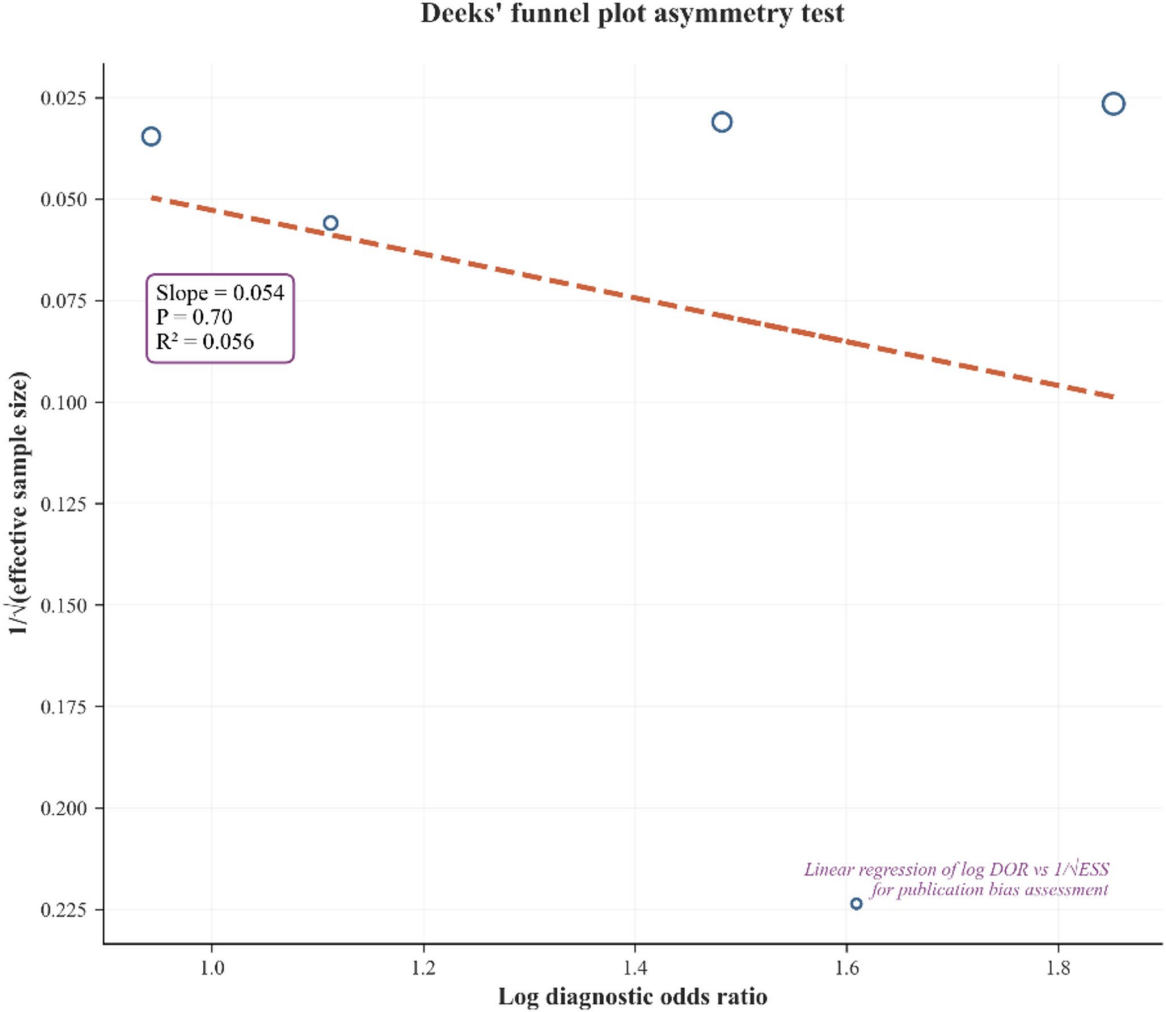
Deeks Funnel Plot

Across studies, the pooled positive likelihood ratio was 1.45 (95% CI 1.17–1.80) (Supplementary Fig. 5), the pooled negative likelihood ratio was 0.37 (95% CI 0.23–0.61) (Supplementary Fig. 6), and the diagnostic odds ratio was 3.95 (95% CI 2.48–6.30) (Supplementary Fig. 7), indicating modest overall discrimination and aligning with the pattern of high sensitivity but low specificity. Applying these LRs in a Fagan nomogram at a 25% pre-test probability (Supplementary Fig. 8) yields a post-test probability of roughly ~ 33% after a positive micro-US and ~ 11% after a negative result, indicating modest probability shifts overall and greater utility for ruling out than ruling in clinically significant disease. Deeks’ funnel plot (Fig. 5) showed no evidence of small-study effects (*p* ≈ 0.70). Goodness-of-fit and residual diagnostics (Supplementary Fig. 9) were acceptable, with residuals centered around zero and no single dominating outlier, supporting the suitability of the bivariate random-effects model.

## Discussion

This systematic review and meta-analysis evaluated 29-MHz micro-ultrasound (micro-US, ExactVu) as a stand-alone index test for classifying clinically significant prostate cancer (csPCa) across five prospective studies. The random-effects model on logit-transformed sensitivity and specificity showed high pooled sensitivity and low pooled specificity, sensitivity 0.84 (95% CI 0.65–0.94) and specificity 0.41 (95% CI 0.25–0.59), with an HSROC consistent with moderate discrimination. Secondary metrics were concordant (PLR 1.45, 95% CI 1.17–1.80; NLR 0.37, 95% CI 0.23–0.61; DOR 3.95, 95% CI 2.48–6.30). On a 25% pre-test probability, Fagan analysis indicated modest probability shifts (~ 33% after a positive micro-US; ~11% after a negative), supporting a triage/rule-out role rather than definitive rule-in.

Heterogeneity was substantial across metrics, and a strong negative correlation between logit-sensitivity and logit-specificity indicated threshold effects as a principal driver of variability. Exploratory meta-regression did not identify robust moderators of sensitivity; spectrum-related covariates were associated with lower specificity but given the small number of studies these findings are hypothesis-generating. Model diagnostics supported the adequacy of the bivariate specification, and Deeks’ test showed no evidence of small-study effects. Overall, micro-US demonstrates high sensitivity and low specificity with modest clinical impact on post-test probability, best positioned as a complementary/triage tool, particularly when mpMRI is unavailable, contraindicated, or delayed, rather than a stand-alone confirmatory test.

The pooled sensitivity of micro-US in our analysis was 84%, indicating that micro-US effectively identifies a substantial proportion of patients with PCa. This finding aligns with the results reported in several of the included studies. For instance, Klotz et al.[9] reported a sensitivity of 94% in a large multicenter registry study including 1,040 patients, while Avolio et al. [15] found a sensitivity of 85% in a prospective cohort of 1,423 men undergoing biopsy. Conversely, Pavlovich et al. [16] reported a lower sensitivity of 60%, which may be explained by the inclusion of early operator learning phases and a more heterogeneous patient population. Our pooled sensitivity is also consistent with previous reviews and meta-analyses. Alghamdi et al. [17], in a meta-analysis comparing multiple US modalities, reported pooled sensitivities for US ranging from 74% to 94%, although their analysis combined micro-US with other US techniques and did not assess micro-US in isolation. Similarly, Ditonno et al. [18] reported favorable sensitivity for micro-US but pooled results across heterogeneous imaging approaches, limiting direct comparisons. The high sensitivity of micro-US can be attributed to its high-frequency (29 MHz) real-time imaging, which enables improved visualization of prostate tissue microstructures. This suggests that micro-US may be an effective tool for early lesion detection, potentially reducing missed clinically significant cancers, especially in settings where MRI is unavailable or contraindicated.

The pooled specificity of micro-US was 41% with study-level estimates spanning (25–59%). This represents **low** specificity, close to chance performance—and argues against using micro-US as a stand-alone **rule-in** test. In contrast, Lughezzani et al. reported a specificity of 60.6%, and Klotz et al. observed a specificity of 22%, reflecting real-world variability [9, 19]. Previous meta-analyses have also highlighted the challenge of achieving high specificity with US-based modalities. In Alghamdi et al.‘s review, specificity values for US varied widely and were generally lower than those reported for MRI, emphasizing the inherent limitations of US-based imaging for tissue characterization [17]. The low specificity implies more **false positives** and potential **unnecessary biopsies**; while standardized scoring (PRI-MUS) and reader experience may improve performance, stricter thresholds that raise specificity generally **reduce sensitivity**, reinforcing a **triage/rule-out** rather than confirmatory role for micro-US.

The pooled PLR of 1.45 and NLR of 0.37 suggest that micro-US has limited value for confirming PCa but may offer moderate utility in ruling it out. These values indicate that patients with PCa are approximately 1.4 times more likely to have a positive micro-US result, while a negative result moderately decreases the probability of disease. The Fagan nomogram, using a pre-test probability of 25%, showed that a positive micro-US result increases the post-test probability to 33%, whereas a negative result reduces it to 11%. Accordingly, the likelihood ratio scatter plot places micro-US in the rule-out-leaning zone, consistent with a “rule-out” function rather than confirmatory use. The pooled DOR of 3.95 reflects modest overall discriminative ability. From a clinical standpoint, these findings indicate that micro-US, while sensitive, lacks the specificity needed to reliably confirm disease and is best positioned as an exclusion tool, particularly in settings where mpMRI is unavailable, contraindicated, or delayed. However, the substantial heterogeneity across studies (I² >80% for sensitivity, specificity and DOR) underscores variability in test performance and highlights the need for standardized operator training, consistent scoring systems (e.g., PRI-MUS) [16, 20], and consideration of disease prevalence when applying micro-US in routine clinical decision-making.

Substantial heterogeneity in diagnostic accuracy was observed and should temper interpretation of the pooled estimates. From a clinical perspective, such variability is unsurprising: imaging performance depends on patient spectrum, reader experience, and local protocols. Our analyses demonstrated a clear **threshold effect**, with a trade-off between sensitivity and specificity that likely reflects differences in positivity criteria (e.g., PRI-MUS cut-offs) and reader behavior. In **subgroup summaries**, sensitivity was broadly stable across strata, whereas **specificity** varied with clinical spectrum: cohorts enriched for higher pre-test risk (such as prior-negative biopsy populations or screening-type case mixes) tended to show lower and more variable specificity, and single-study strata often combined very high sensitivity with very low specificity, consistent with threshold/spectrum phenomena and warranting cautious interpretation. In **meta-regression**, no study characteristic consistently shifted sensitivity, while indicators of enriched spectrum were associated with lower specificity; by contrast, factors such as sample size, center type, biopsy approach or guidance, reference standard, and geography did not explain residual variability. Taking them together, these findings suggest that heterogeneity is driven predominantly by **thresholding and spectrum** rather than by any single procedural detail. Standardizing PRI-MUS thresholds, reinforcing reader training, and clearly defining target populations may improve calibration; clinically, micro-US is most defensible as a **triage/rule-out** tool in pathways where rapid, accessible assessment is needed or mpMRI access is limited, with explicit acknowledgment of the sensitivity–specificity trade-off.

### Comparison with mpmri: diagnostic accuracy and feasibility

Our pooled micro-US performance (sensitivity 0.84; specificity 0.41) should be interpreted in the context of contemporary mpMRI. In the paired-design PROMIS study using template mapping as the reference, mpMRI achieved high sensitivity for clinically significant cancer (≈ 93%) with lowest specificity (≈ 41%), broadly like the specificity we observed for micro-US but with higher sensitivity overall [21]. In larger evidence syntheses, a 2019 meta-analysis of 29 prospective mpMRI studies (8,503 men) reported pooled sensitivity of 0.87 and specificity of 0.68 for suspected prostate cancer, indicating that mpMRI typically operates on a more favorable sensitivity–specificity frontier than micro-US in our analysis [22]. Current guidelines therefore endorse an MRI-first pathway before biopsy in biopsy-naïve men, reflecting both diagnostic performance and the ability of mpMRI to guide targeted sampling.

Feasibility considerations, however, may favor complementary roles. mpMRI requires specialized scanners, protocols, and radiology expertise, with longer scheduling and higher costs, whereas micro-US is bedside, real-time, contrast-free, and can be integrated into the same-day diagnostic visit. Against this, our meta-analysis shows that micro-US specificity is low, implying more false positives if used as a stand-alone rule-in test. A pragmatic interpretation is that micro-US could serve as a rapid triage/rule-out adjunct where mpMRI is unavailable, contraindicated, or delayed, or as an access-expanding gatekeeper to MRI in resource-constrained settings. Determining the optimal sequencing or combination (e.g., micro-US–guided targeting with or without MRI) will require prospective head-to-head or combined-strategy studies powered for clinically significant endpoints [23].

### Cost and resource implications

Formal, peer-reviewed economic evaluations directly comparing stand-alone micro-ultrasound with mpMRI are currently scarce. By contrast, multiple analyses suggest that mpMRI-first pathways are cost-effective versus systematic TRUS biopsy alone because they reduce unnecessary biopsies and overdiagnosis. For example, a Singapore health-system model found pre-biopsy MRI strategies to be cost-effective relative to TRUS-only pathways across plausible willingness-to-pay thresholds, primarily by improving detection of clinically significant cancer and avoiding low-yield biopsies [24]. In settings where scanner capacity is constrained, abbreviated/shorter MRI protocols have also been reported as highly cost-effective compared with full mpMRI while maintaining diagnostic performance, further supporting MRI-first pathways from an economic standpoint. Representative unit-cost data used in screening and diagnostic models (e.g., UK analyses drawing on NICE resource estimates) place mpMRI as a relatively high-cost diagnostic compared with ultrasound-based approaches, reinforcing that per-examination costs and infrastructural needs are materially greater for MRI [25].

Micro-ultrasound, as an ultrasound-based, contrast-free, point-of-care modality, is inherently less resource-intensive in terms of equipment, scheduling, and on-site logistics; early prospective trials comparing micro-US–guided pathways with MRI-based strategies suggest potential for wider accessibility and lower direct costs, though these signals come largely from feasibility and non-economic reports to date [26]. Taken together, the current evidence supports mpMRI as cost-effective versus TRUS-only pathways, while the cost-effectiveness of micro-ultrasound—either as a stand-alone alternative or as a triage gatekeeper to mpMRI—remains an open question. Prospective economic evaluations that quantify quality-adjusted life-years, downstream biopsy utilization, and incremental cost-effectiveness ratios for micro-US–first or combined micro-US/mpMRI strategies are needed to define the most efficient deployment across health-system contexts.

### Strengths and limitations

This work is, to our knowledge, among the first pooled diagnostic-test-accuracy syntheses focused exclusively on 29-MHz micro-ultrasound as a stand-alone index test for classifying clinically significant prostate cancer (csPCa) in prospective cohorts. We limited inclusion to studies using histopathology obtained in the same diagnostic episode and a standardized platform (ExactVu), which improves comparability while recognizing remaining spectrum and threshold differences. We applied a random-effects pooling of logit-transformed sensitivity and specificity with an HSROC representation and pre-specified thresholds with pre-specified thresholds (primary PRI-MUS ≥ 3, secondary ≥ 4), presented HSROC curves, and conducted comprehensive diagnostics (leave-one-out influence, goodness-of-fit, and residual checks). Subgroup summaries and meta-regression were used to explore heterogeneity, and sensitivity analyses (e.g., excluding the spectrum-selected cohort and the randomized guidance trial) assessed robustness. Finally, we quantified clinical impact with likelihood ratios, DOR, and Fagan nomograms, providing decision-relevant interpretation for real-world pathways where rapid triage or limited mpMRI access is a concern.

Several limitations merit consideration. The evidence base remains **small** (five prospective studies), which limits precision and renders **subgroup** and **meta-regression** results **exploratory** rather than confirmatory. **Between-study heterogeneity** was substantial across all metrics and appears driven chiefly by **thresholding** (differences in PRI-MUS cut-offs and reader behavior) and **clinical spectrum** (e.g., enrichment by prior-negative biopsy or MRI-negative pathways), with one included randomized **guidance** trial contributing design diversity; although leave-one-out checks suggested no single study dominated the results, pooled estimates should be interpreted cautiously. **Generalizability** may be constrained because most cohorts were from experienced, often academic centers using a single platform (ExactVu), and performance can vary with **operator training** and learning curve. Despite using **histopathology** as the reference standard, variations in **biopsy approach** (transrectal vs. transperineal), **sampling strategy** (systematic, targeted, or combined), **guidance modality**, and **csPCa definitions** (e.g., ISUP ≥ 2 vs. alternatives) may introduce verification and classification differences that our study-level moderators could not fully capture. Although **Deeks’ test** did not indicate small-study effects, the **low power** inherent to few studies means selective reporting or publication bias cannot be excluded. Future research should prioritize **multicenter**,** prospective DTA** designs with **pre-specified PRI-MUS thresholds**, explicit **blinding**, and **uniform verification** irrespective of test result; include **community settings**; perform **head-to-head** and **combined-strategy** evaluations with mpMRI; and report **decision-curve** and **cost-effectiveness** outcomes to clarify the most efficient role for micro-US in routine pathways.

### Conclusion

Micro-US shows **high sensitivity but low specificity** for **classifying clinically significant prostate cancer**, resulting in **modest** overall discrimination and only modest shifts in post-test probability. In practice, it is best used as a **triage/rule-out adjunct**, especially when mpMRI is unavailable, contraindicated, delayed, or when same-day assessment is needed, rather than as a stand-alone rule-in test; its performance is sensitive to **threshold** and **clinical spectrum**, reinforcing the need for standardized **PRI-MUS** application and reader training.

## Supplementary Information

Below is the link to the electronic supplementary material.

Supplementary Material 1

Supplementary Material 2

Supplementary Material 3

Supplementary Material 4

Supplementary Material 5

Supplementary Material 6

Supplementary Material 7

Supplementary Material 8

Supplementary Material 9

Supplementary Material 10

## Data Availability

All data were derived from published articles; extraction sheets and analytic code are available upon reasonable request.
