# Supplementary material for: Standalone 29-MHz micro-ultrasound for classifying clinically significant prostate cancer: a systematic review and diagnostic test accuracy meta-analysis of prospective studies
Source: Abdom Radiol (NY). 2025 Oct 31;51(6):2979–92. doi: 10.1007/s00261-025-05218-x (PMC13109128; doi:10.1007/s00261-025-05218-x)
Supplement: Supplementary file 1 — Supplementary Material 1 [file 261_2025_5218_MOESM1_ESM.docx]

| Supplementary Table 1. Search strategy for each database | |  |
| --- | --- | --- |
| Database | **Search strategy** | **Number of results** |
| PubMed | ((Prostate Cancer*[Title]) OR (prostatic neoplasm*[Title])) AND ((ultrasound[Title]) OR (ultrasonography[Title]) OR (29 MHz micro-ultrasound[Title]) OR (ExactVu[Title])) | 1441 |
| Embase | ('prostate cancer'/exp OR 'prostate cancer' OR (('prostate'/exp OR prostate) AND ('cancer'/exp OR cancer)) OR 'prostatic neoplasm'/exp OR 'prostatic neoplasm' OR (prostatic AND ('neoplasm'/exp OR neoplasm))) AND ('29 mhz micro-ultrasound' OR (29 AND mhz AND ('micro ultrasound'/exp OR 'micro ultrasound')) OR 'exactvu'/exp OR exactvu) | 151 |
| Scopus | ( ( Prostate Cancer ) OR ( prostatic neoplasm ) ) AND ( ( ultrasound ) OR ( ultrasonography ) OR ( 29 MHz micro-ultrasound ) OR ( ExactVu ) ) | 1876 |
| Web of Science | ( ( Prostate Cancer ) OR ( prostatic neoplasm ) ) AND ( ( ultrasound ) OR ( ultrasonography ) OR ( 29 MHz micro-ultrasound ) OR ( ExactVu ) ) | 2351 |
