# Supplementary figures and images for: Standalone 29-MHz micro-ultrasound for classifying clinically significant prostate cancer: a systematic review and diagnostic test accuracy meta-analysis of prospective studies
Source: Abdom Radiol (NY). 2025 Oct 31;51(6):2979–92. doi: 10.1007/s00261-025-05218-x (PMC13109128; doi:10.1007/s00261-025-05218-x)

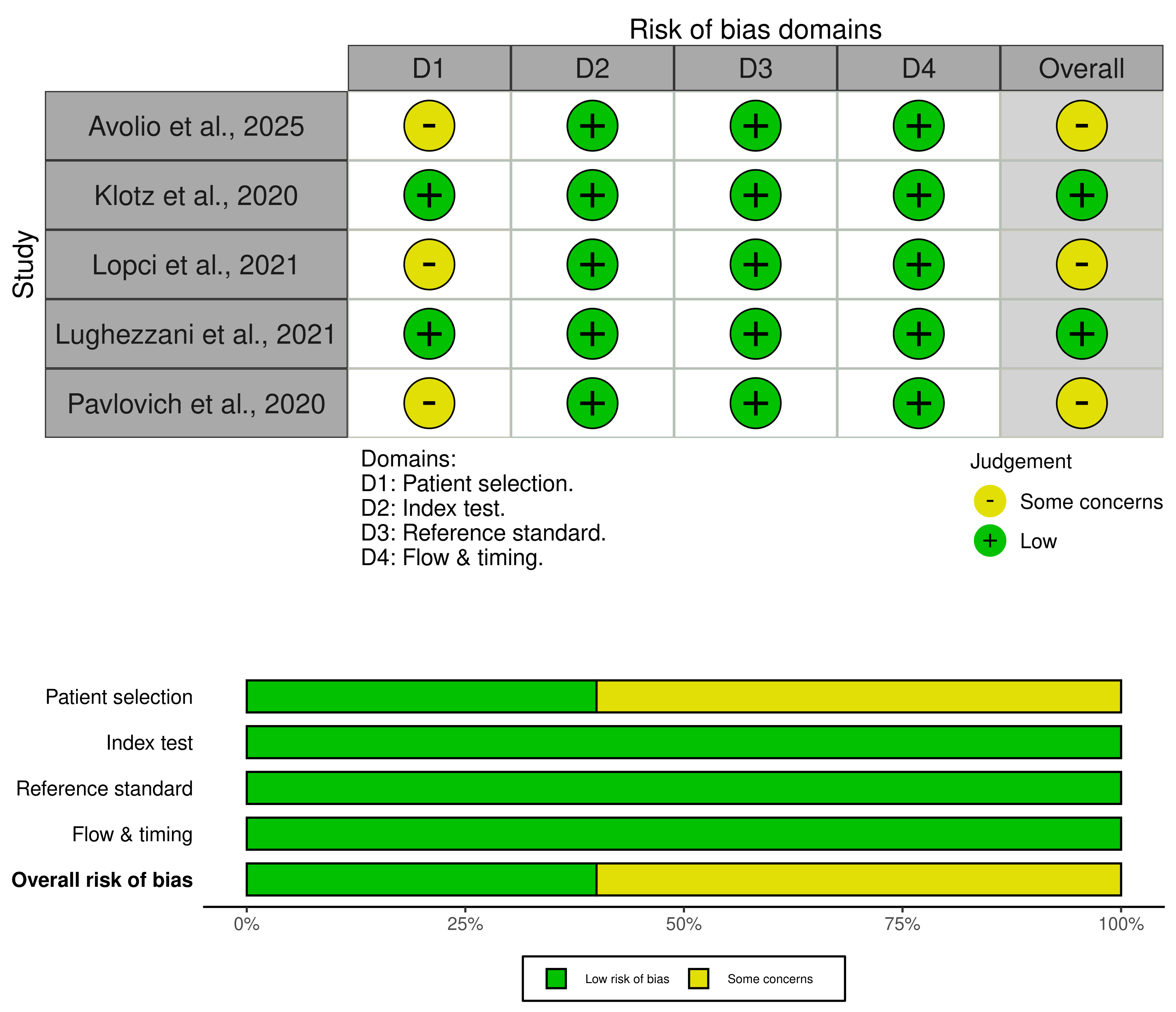

Supplement: Supplementary file 2 — Supplementary Material 2 [file 261_2025_5218_MOESM2_ESM.png]

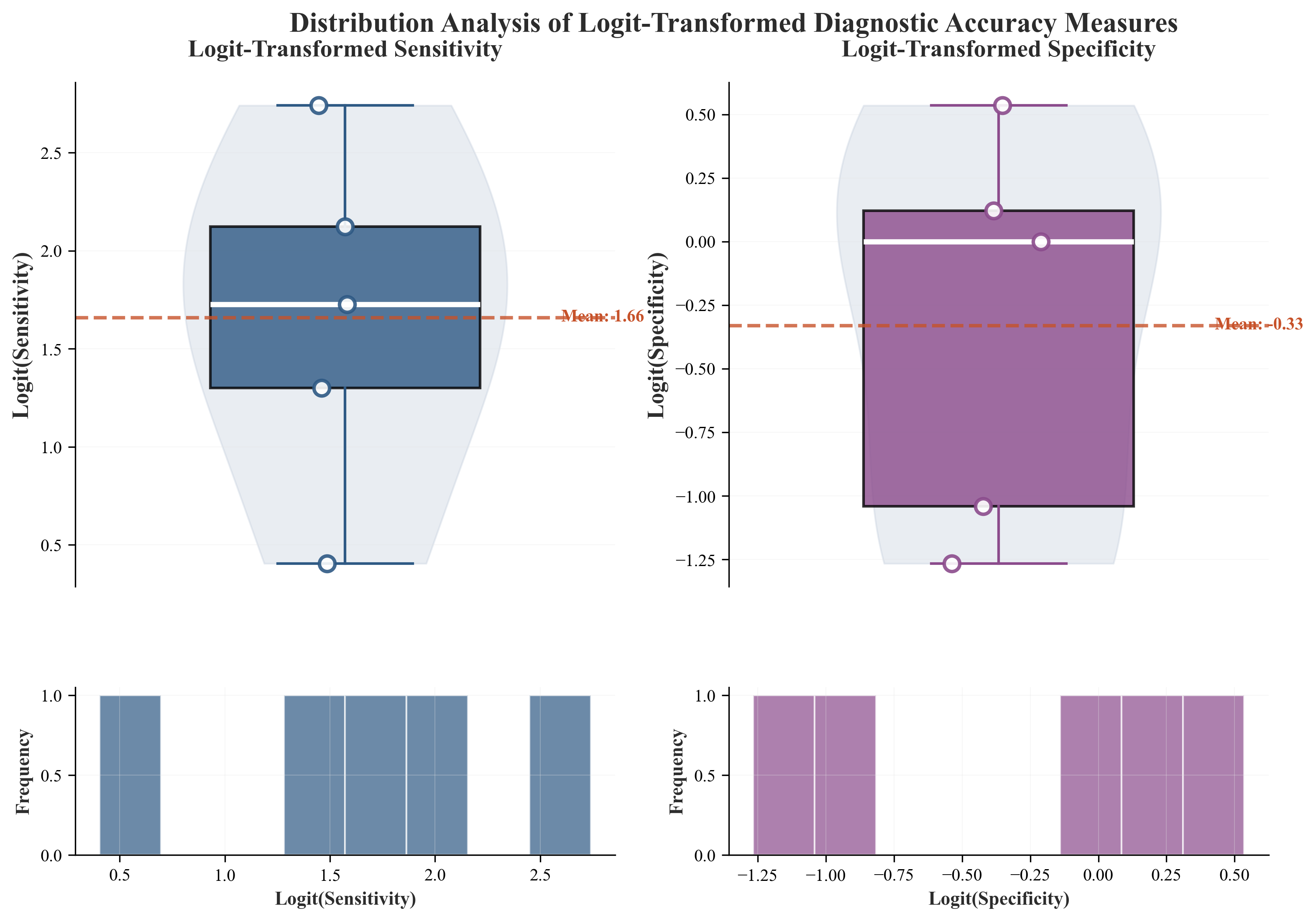

Supplement: Supplementary file 3 — Supplementary Material 3 [file 261_2025_5218_MOESM3_ESM.png]

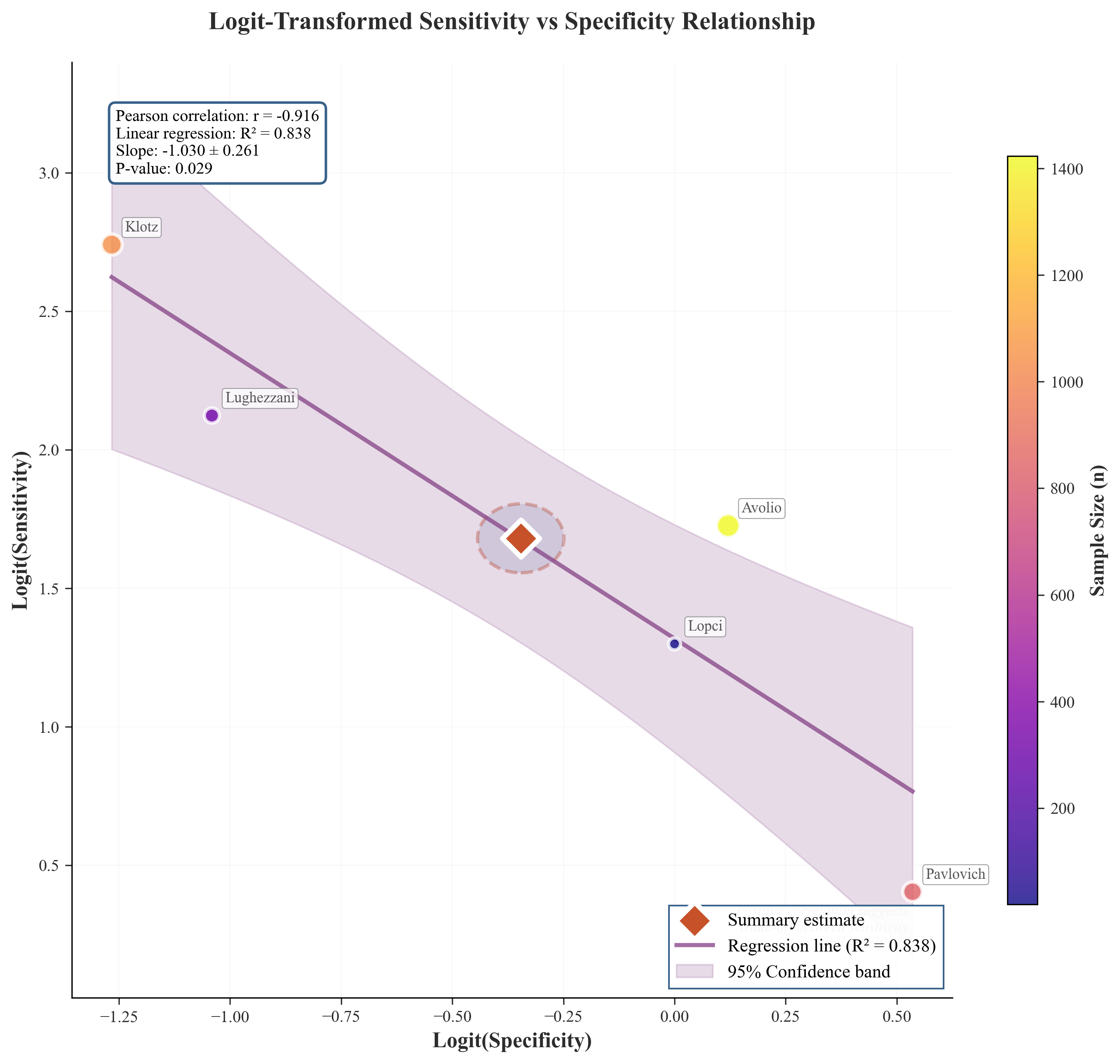

Supplement: Supplementary file 4 — Supplementary Material 4 [file 261_2025_5218_MOESM4_ESM.png]

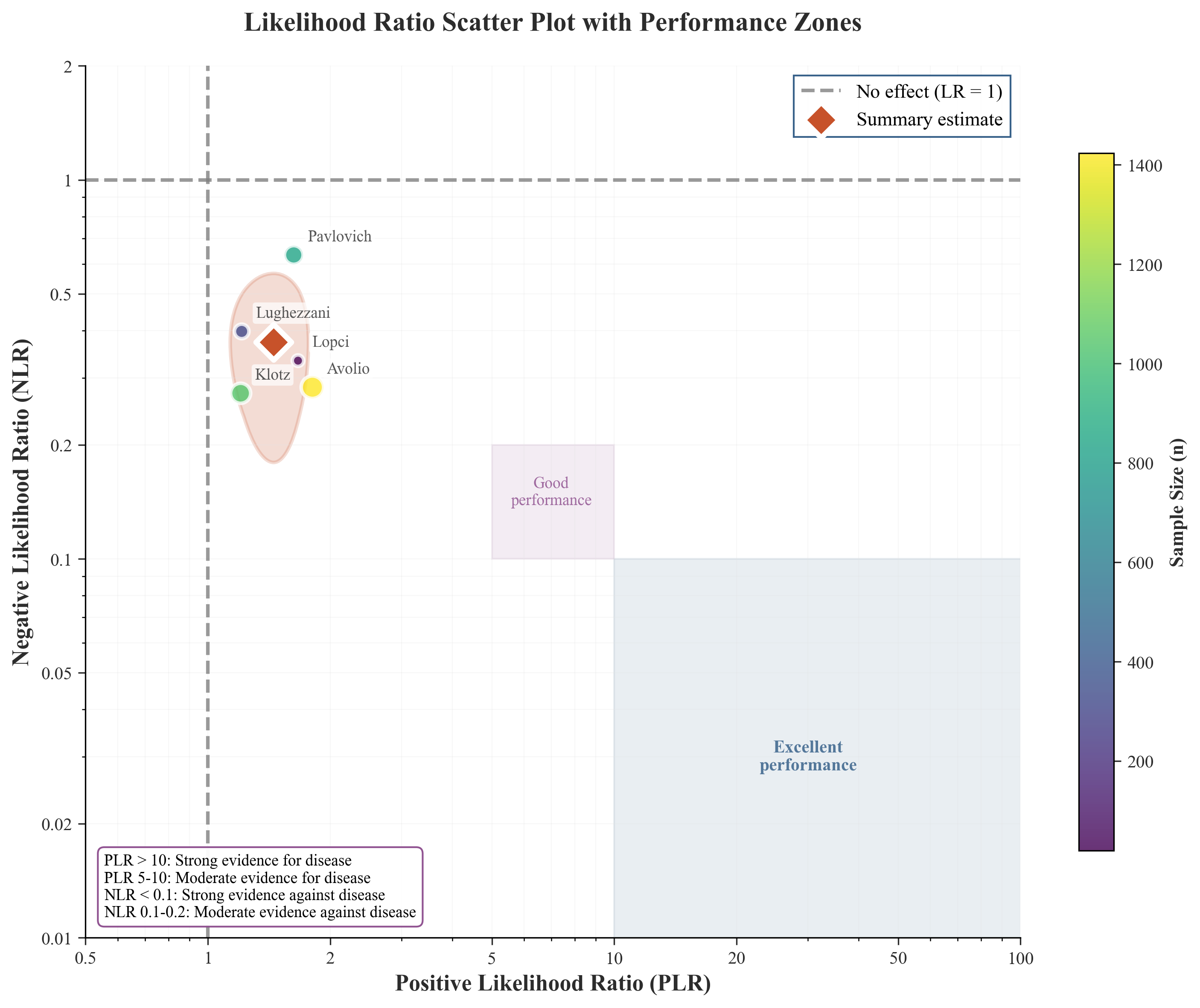

Supplement: Supplementary file 5 — Supplementary Material 5 [file 261_2025_5218_MOESM5_ESM.png]

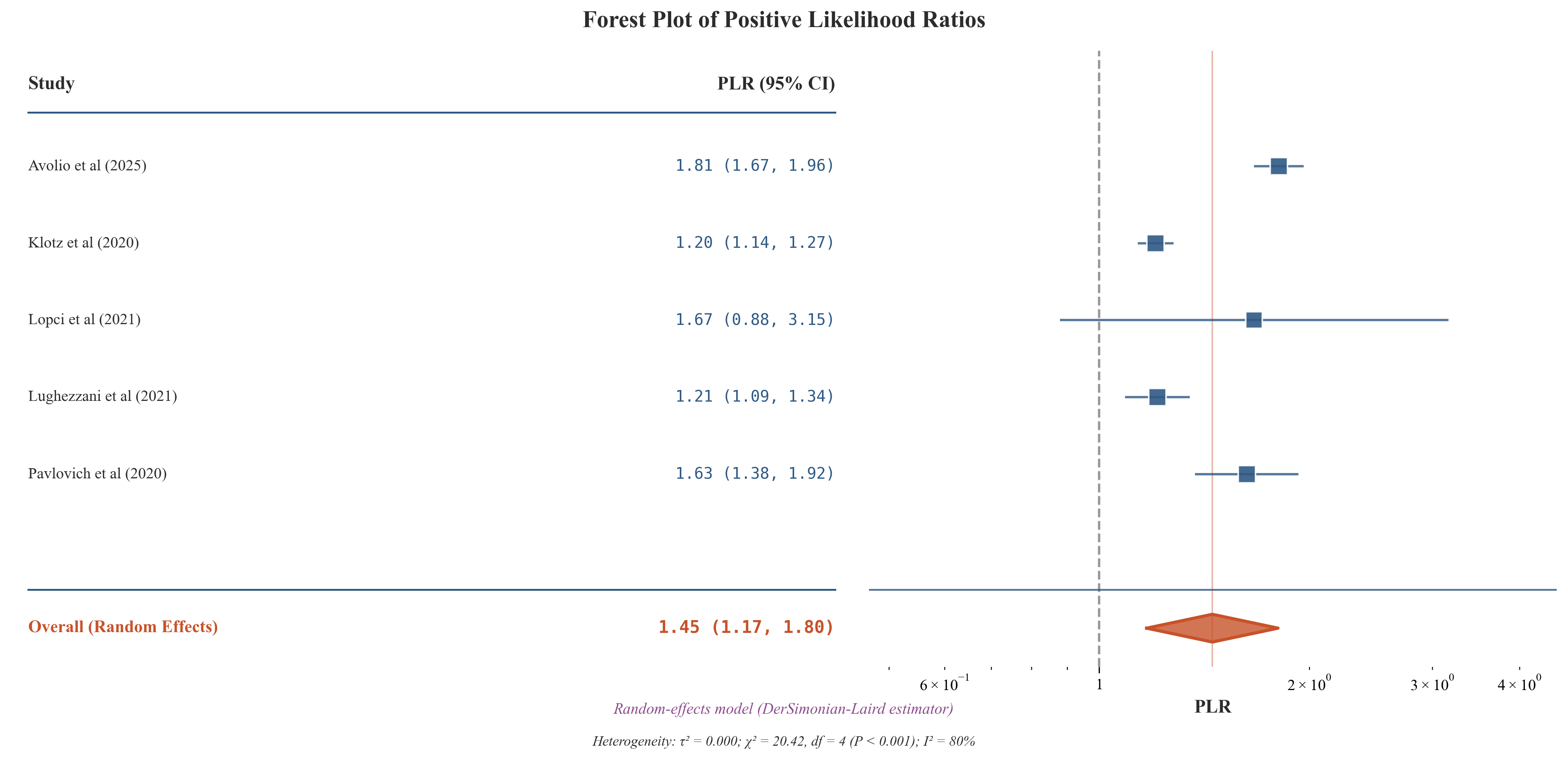

Supplement: Supplementary file 6 — Supplementary Material 6 [file 261_2025_5218_MOESM6_ESM.png]

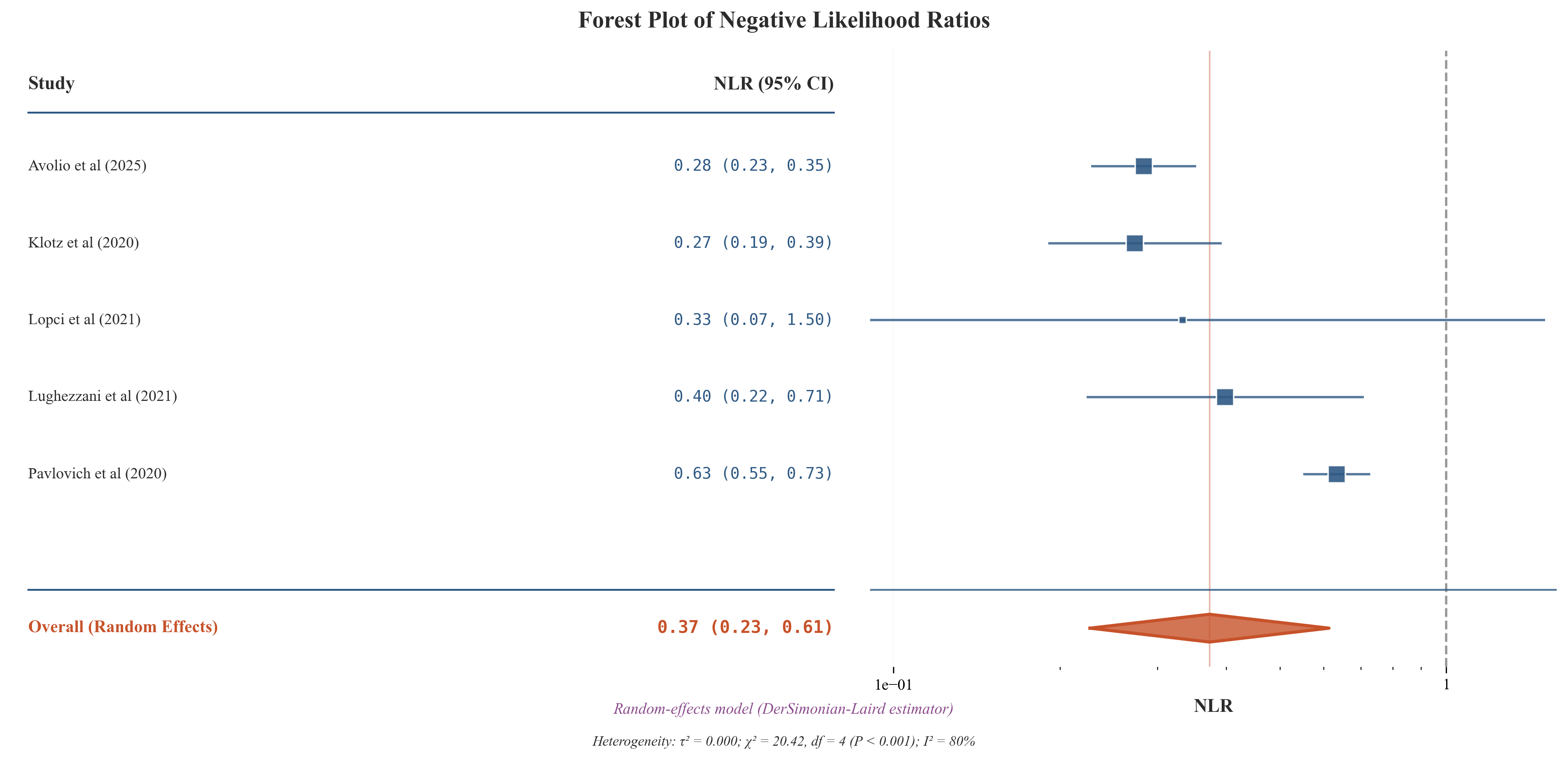

Supplement: Supplementary file 7 — Supplementary Material 7 [file 261_2025_5218_MOESM7_ESM.png]

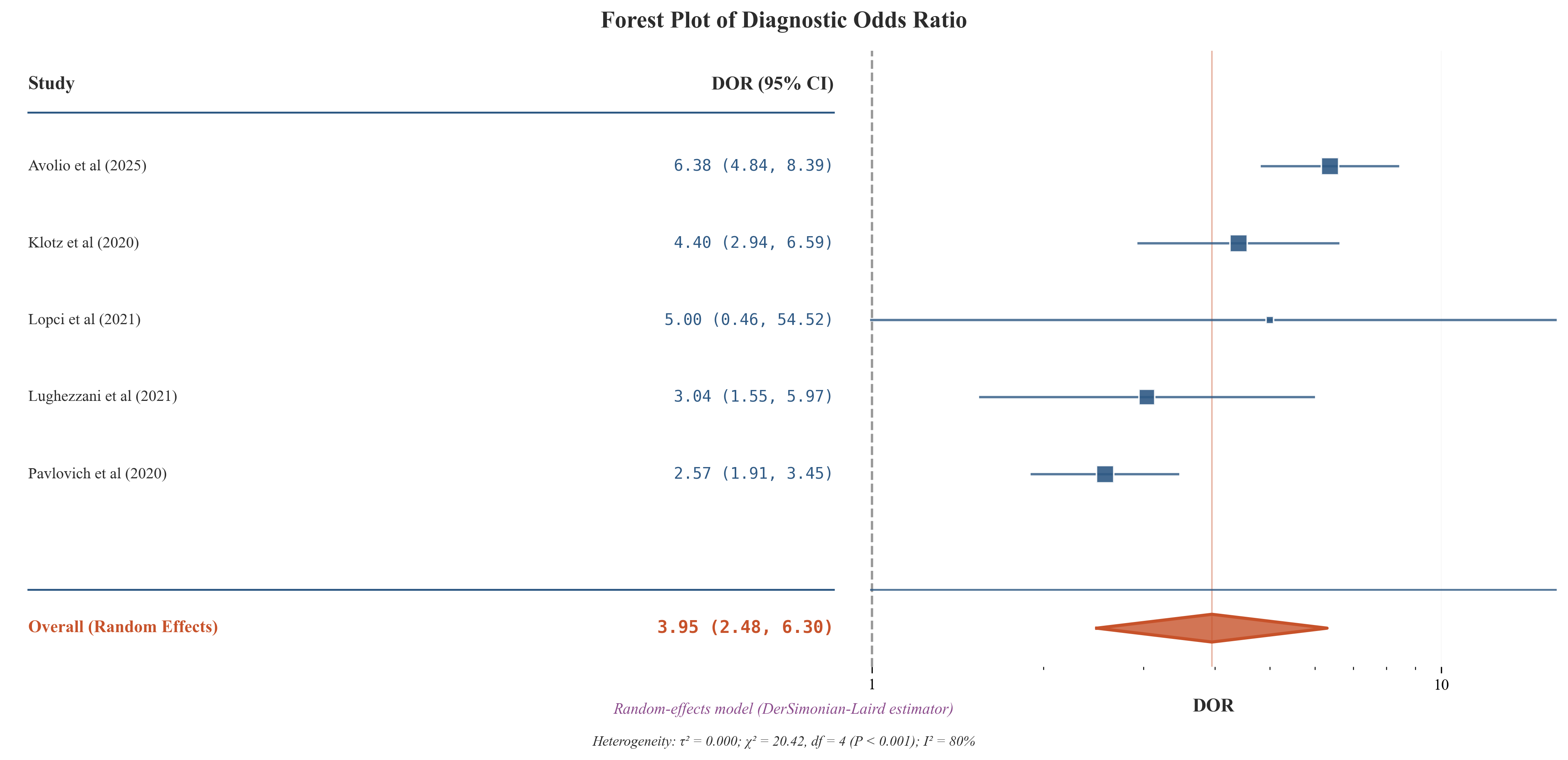

Supplement: Supplementary file 8 — Supplementary Material 8 [file 261_2025_5218_MOESM8_ESM.png]

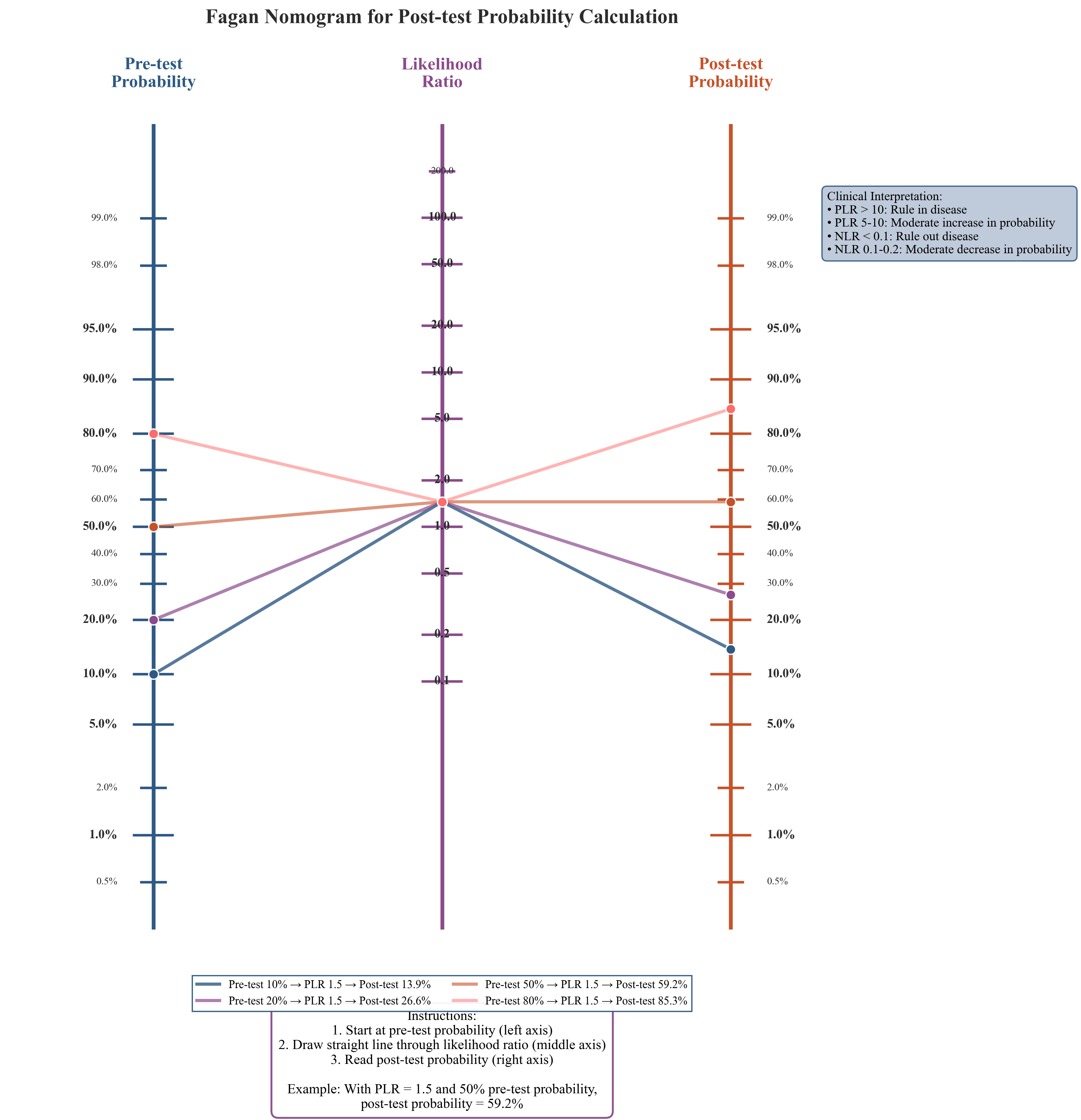

Supplement: Supplementary file 9 — Supplementary Material 9 [file 261_2025_5218_MOESM9_ESM.png]

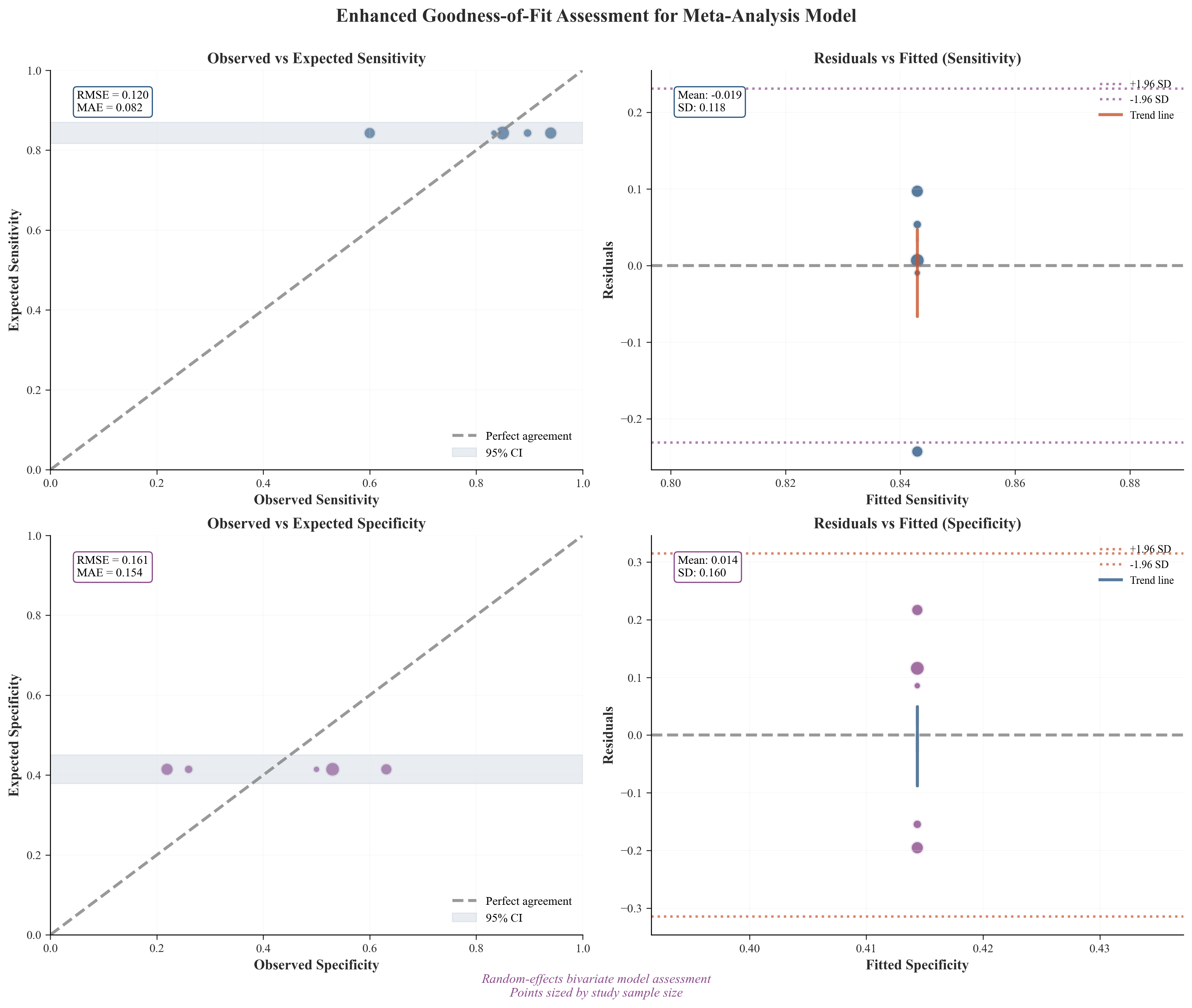

Supplement: Supplementary file 10 — Supplementary Material 10 [file 261_2025_5218_MOESM10_ESM.png]
